# A predictive computational model of the kinetic mechanism of stimulus-induced transducer methylation and feedback regulation through CheY in archaeal phototaxis and chemotaxis

**DOI:** 10.1186/1752-0509-4-27

**Published:** 2010-03-18

**Authors:** Stefan Streif, Dieter Oesterhelt, Wolfgang Marwan

**Affiliations:** 1 Max Planck Institute for Dynamics of Complex Technical Systems, Molecular Network Analysis Group, Sandtorstr. 1, 39106 Magdeburg, Germany; 2 Max Planck Institute of Biochemistry, Department of Membrane Biochemistry, Am Klopferspitz 18, 82152 Martinsried, Germany

## Abstract

**Background:**

Photo- and chemotaxis of the archaeon *Halobacterium salinarum *is based on the control of flagellar motor switching through stimulus-specific methyl-accepting transducer proteins that relay the sensory input signal to a two-component system. Certain members of the transducer family function as receptor proteins by directly sensing specific chemical or physical stimuli. Others interact with specific receptor proteins like the phototaxis photoreceptors sensory rhodopsin I and II, or require specific binding proteins as for example some chemotaxis transducers. Receptor activation by light or a change in receptor occupancy by chemical stimuli results in reversible methylation of glutamate residues of the transducer proteins. Both, methylation and demethylation reactions are involved in sensory adaptation and are modulated by the response regulator CheY.

**Results:**

By mathematical modeling we infer the kinetic mechanisms of stimulus-induced transducer methylation and adaptation. The model (deterministic and in the form of ordinary differential equations) correctly predicts experimentally observed transducer demethylation (as detected by released methanol) in response to attractant and repellent stimuli of wildtype cells, a *cheY *deletion mutant, and a mutant in which the stimulated transducer species is methylation-deficient.

**Conclusions:**

We provide a kinetic model for signal processing in photo- and chemotaxis in the archaeon *H. salinarum *suggesting an essential role of receptor cooperativity, antagonistic reversible methylation, and a CheY-dependent feedback on transducer demethylation.

## Background

The archaeon *Halobacterium salinarum *swims by rotation of a semi-rigid right-handed flagellar bundle [1]. Each flagellar filament of the bundle extends the axis of a rotary motor to passively transduce the mechanical energy generated by the motor to the medium. Motors are anchored in the cell membrane and driven by ATP [2]. Cells swim back and forth by switching the sense of flagellar rotation from clockwise to counterclockwise and *vice versa *[1,3]. In adapted or unstimulated cells, switching occurs spontaneously. Active swimming and motor switching is superimposed with passive Brownian motion of cell body and flagellar bundle [4]. By the resulting random swimming paths, the cells explore their environment until they encounter a stimulus. Sensory stimulation of the photo- or chemoreceptors transiently modulates the probability of motor switching, resulting in a movement of the cell towards more favorable regions in the environment [5,6]. Rather than sensing absolute stimulus strengths, cells respond to relative changes by adapting to any stimulus of constant intensity [7,8].

Several lines of evidence suggest that in bacteria as well as in archaea this adaptation is at least partially caused by reversible methylation of methyl-accepting taxis proteins [7,9,10]. Proteins of this family, also called transducers, may act as sensory receptors for a specific stimulus, or they may bind to a specific, but different receptor protein to transduce the activation state of this respective receptor and relay it to the autophosphorylating histidine kinase CheA [11-13]. CheA phosphorylates the CheY protein, which is required for flagellar motor switching [14], and the phosphorylation rate is thought to be stimulus-dependent.

**Figure 1:**
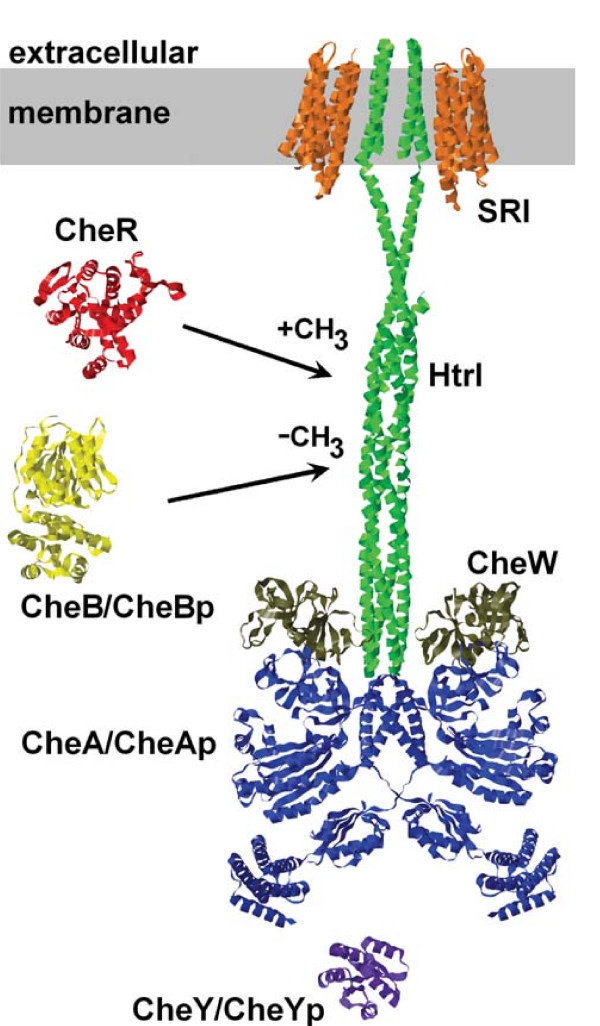
**The structures of the components of the photosensory system of H. salinarum**. The figure shows the structures of the components of the receptor-transducer-CheW-CheA (R-TWA) complex (stoichiometry SRI:HtrI:CheW:CheA = 2:2:2:2) and of CheY, CheB, and CheR. The membrane embedded light-receptor SRI (orange) interacts with the membrane embedded transducer HtrI (green) and the signaling state is transferred to the histidine kinase CheA (blue) which is bound via the scaffold protein CheW (brown) to the signaling domain of HtrI. An active signaling state of HtrI enhances autophosphorylation of CheA, and the phosphate group is then transferred to the response regulator CheY (purple). The methyl-transferase CheR (red; +CH_3_) and the methyl-esterase CheB (yellow; -CH_3_) bring about adaptation by reversible methylation. Protein structures were obtained by homology modeling and the arrangement of the quaternary structure is based on the spatial organization of the *Escherichia coli *chemotaxis system proposed by [77,78] and of the SRII and HtrII interaction in the archeaon *Natronomonas pharaonis *[29]. The arrangement is not meant to imply that the domains or proteins are oriented or contact each other in the depicted manner.

The genome of *H. salinarum *encodes 18 orthologous methyl-accepting taxis proteins (also called Htr's for *H*alobacterial *tr*ansducers) [15]. While sensing and transmembrane regions of these orthologs are quite diverse, the cytoplasmic domains share a high degree of similarity. The diversity allows monitoring different types of cellular and environmental parameters: light, oxygen, proton motive force, amino acids, temperature, and presumably others. The similarity of the cytoplasmic domains of the transducers guarantees sensory integration by CheA of all of these stimuli [16,17]. The transducers physically interact with CheA and the scaffolding proteins CheW1 and CheW2 [18] to form R-TWA complexes (see Figure 1), and the transducers localize in clusters at the cell poles [19] where the photosensory sensitivity is restricted to [20].

With the help of two sensory rhodopsins, SRI and SRII, *H. salinarum *senses orange, uv and blue light (see [7] and references therein). The sensory rhodopsins directly act as photoreceptors through physical association with their cognate methyl-accepting proteins (HtrI and HtrII) [21-24]. The transducers form stable complexes with the sensory rhodopsins [22,24-26] and the cytoplasmic domain of the transducer is essential for the functional interaction of the two molecules [21]. After photoexcitation, which causes photoisomerization of the covalently bound retinal chromophor (all-*trans *to 13-*cis*), a sensory rhodopsin molecule proceeds through a sequence of metastable intermediates and finally returns to the initial state through re-isomerization of the chromophor [27,28]. This so-called photocycle is thermodynamically driven by part of the energy of the absorbed photon. The conformational changes of the sensory rhodopsin molecule caused by photocycling are transduced to the complexed Htr molecule and regulate CheA activity [29,30]. The enzymatic signaling mechanism does not involve changes in the membrane potential [20]. Differential regulation of motor switching is thought to be caused by differential regulation of the CheA kinase activity. Orange and uv light are sensed by SRI, suppressing or inducing motor switching respectively, and blue light is sensed by SRII, inducing motor switching [7].

Most halobacterial transducer proteins carry 1 to 3 potential methylation sites (glutamyl-residues) per molecule [31]. As in bacteria [9], the methyl-transferase CheR transfers the methyl-group of S-adenosyl-methionine to a glutamate residue of the transducer signaling domain [31-33]. After incubating cells with ^3^H-labeled methionine, transducer methylation is detected by fluorography [34-38].

Demethylation is catalyzed by the (C-terminal) methyl-esterase domain of CheB [31-33]. In *E. coli*, phosphorylation (by CheA) of the N-terminal, CheY-like domain of CheB increases methyl-esterase activity [39]. In *H. salinarum*, the CheY-like domain of CheB is also present [33], but it is not known whether it becomes phosphorylated in a stimulus-dependent manner or whether CheB-phosphorylation modulates methyl-esterase activity *in vitro *or *in vivo*.

**Figure 2:**
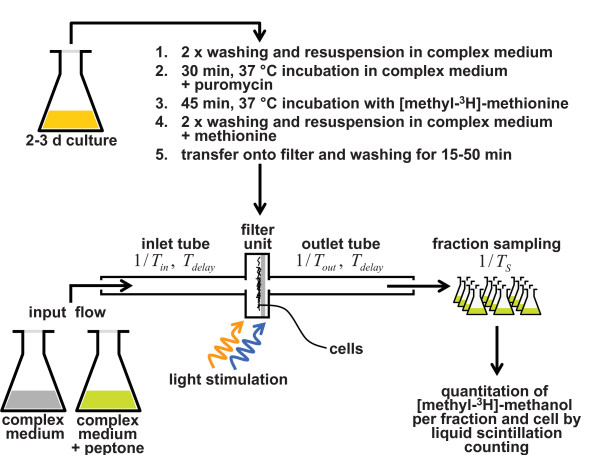
**Illustration of the flow assay used for measuring transient demethylation rates**. The assay performed by [34-36,38] to detect demethylation rates of *Halobacterium salinarum *is a slight variation of an assay originally used for *E. coli *[40]. Cells are incubated in the presence of [methyl-^3^H]-methionine and puromycine (inhibitor of protein biosynthesis) to label the transducer methyl-groups. Cells are then transferred to the filter unit and chemotaxis stimuli are applied by switching between complex medium (without peptone) and complex medium with peptone. Phototaxis stimuli are applied by exposing the cells on the filter to light. Fractions are collected with fixed sampling rate *T*_*S *_and are subsequently processed by liquid scintillation counting to determine the release of volatile [methyl-^3^H]-methionine as [methyl-^3^H]-methanol. Due to turbulent flow and mixing kinetics, the measurements and the chemo-stimuli are subject to certain dynamics (time constants *T*_*in*_, *T*_*out*_, and *T*_*delay*_) that extend the time course of the methanol release [35,36]. For further details see Methods.

Transducer demethylation results in formation of methanol [35] which can be quantitatively measured in a time-resolved manner with a so-called flow-assay (see Figure 2; [35,40]). Exposure of *H. salinarum *to any step-like stimulus that causes taxis results in a transient release of methanol, no matter whether the stimulus induces or represses motor switching [34-38,41,42].

The methanol release patterns observed in *H. salinarum *are similar in *Bacillus subtilis *where the phenomenon is explained by the fact that transducers contain methylation sites that were shown to be functionally different [43,44]: one site is demethylated upon stimulation with attractant, another site is demethylated upon stimulation with repellent. Selective methylation has been shown to be regulated by CheY [45] and to depend on transducer/receptor conformation [46].

In *B. subtilis*, the transducer deamidase CheD regulates activity of the CheY-phosphatase CheC [47] and the interplay of CheC and CheD is thought to provide an additional and methylation-independent adaptation system [10,47]. Deletion mutant studies also suggest that CheC and CheD are possibly involved in a coordination of selective methylation in *B. subtilis *(see [10] and references therein). *cheC *and *cheD *orthologs are also found in *H. salinarum *[15,48,49], but their function is not clear.

In *B. subtilis *and in *H. salinarum *the demethylation reaction as measured in a flow assay cause adaptation to stimuli of constant strength, no matter whether a respective stimulus is attractive or repulsive [35,43]. Presumably, differential methylation resets the signaling activity of the transducers to the pre-stimulus level, resulting in sensory adaptation of the cell, although this has not been directly shown. Two mechanisms of stimulus-controlled transducer methylation seem possible: the stimulus-activated transducer molecule may be methylated/demethylated to reset its signaling activity to the pre-stimulus level, or other, i. e. non-stimulated transducers may be methylated/demethylated through a coupling mechanism or feedback loop to attenuate their constant signaling output. Because specific photoactivation of a HtrI mutant in which the methylation sites have been deleted, nevertheless causes both, sensory adaptation and methanol release, coupling between stimulated and unstimulated transducers in terms of reversible methylation seems obvious [38].

A model of the halobacterial motor switch cycle quantitatively reproduces measurements on spontaneous and light stimulus-induced motor switching [50]. In this model, the input to the motor switch is generated by a simple model of the sensory excitation and adaptation processes. Coupling of both models is sufficient to reproduce quantitative data on motor switching, but the simple model of excitation and adaptation does not explain reversible transducer methylation. Here we provide a predictive computational model of sensory adaptation and excitation based on experimental findings on reversible transducer methylation in wildtype and mutant cells.

## Results

### Modeling concepts

**Figure 3:**
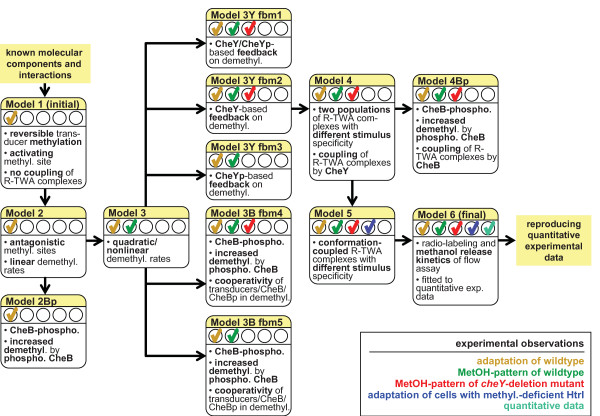
**Overview of models and model extensions used to derive the final, quantitative model**. Starting with the initial Model 1, the quantitative (final) Model 6 was derived step by step keeping the model structure as simple as possible. Models were extended by additional mechanisms/features (see box of each model) to predict finally all of the experimental observations (see legend at the lower right corner). Experimental observations that a model can predict are indicated by the color coded checkmarks ✓. Further properties of the models and the rate equations are given in the Text and in the simulation scripts (Additional file 3).

**Figure 4:**
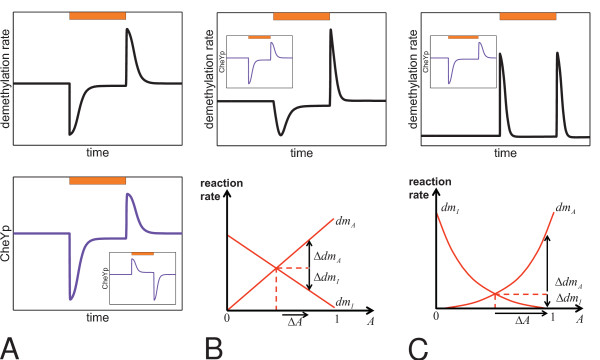
**Transient transducer demethylation rates and CheYp levels for different kinetic mechanisms and models**. Orange bars indicate the time intervals of stimulation with attractant orange light. **(A) **The CheYp-level adapts (bottom) in Model 1, but only *E. coli*-type methanol release patterns (top) are obtained, irrespective of the chosen parameters. **(A inset) **A variation of Model 1 that demonstrates the inverted repellent response of mutant cells to normally attractant orange light [55]. **(B) **The CheYp-level adapts (top, inset) in Model 2, but only *E. coli*-type methanol release patterns (top) are produced on changes in activity (Δ*A*): if reaction rates *dm*_*A *_and *dm*_*I *_depend linear on activity *A*, changes in the rates (Δ*dm*_*A*_, Δ*dm*_*I*_) almost compensate each other and no net increase of demethylation occurs (bottom). **(C) **The CheYp-level adapts (top, inset) in Model 3 and *Halobacterium*-type methanol release patterns (top) can occur in Model 3 with antagonistic methylation and transducer modification rates that depend quadratic on *A *(bottom).

By iterative cycles of mathematical modeling (Figure 3), parameter estimation, and comparison of simulation and experimental data, we obtained a quantitative model of excitation and adaptation in halobacterial phototaxis and chemotaxis. This model consistently explains physiological excitation, adaptation, and experimental data on transient transducer demethylation rates. The model is based on the following experimental observations:

• In wildtype cells, transducer demethylation always increases transiently when previously adapted cells are stimulated, no matter whether an attractant or repellent is set on or off [34-38]. The duration of the transient transducer demethylation approximately correlates with the kinetics of behavioral adaptation of the cells [35]. Transient transducer demethylation and CheYp concentration negatively correlate in response to attractant stimulation and positively correlate in response to repellent stimulation (*Halobacterium*-type pattern, e. g. Figure 4C top).

• In a *cheY *deletion mutant, transducer demethylation decreases transiently upon stimulation with attractants, and increases transiently upon stimulation with repellents [38] (*E. coli*-type pattern, e. g. Figure 4B top).

• A mutant in which all putative methylation sites have been removed from the transducer HtrI still shows wildtype transducer demethylation patterns and sensory adaptation, even if stimulated through the methylation-deficient transducer HtrI, indicating that methyl-groups are turned-over on other transducers that did not receive or transduce the stimulus [38].

The final mathematical model (Model 6; model equations, parameter values, and notation see Methods and Additional file 1) was derived step by step (see Figure 3), keeping the model structure (components and interactions) as simple as possible. The model predicts that

• transducers are organized in clusters (oligomers, multimers) of conformationally coupled molecules

• a cluster is composed of transducers with different input specificity in terms of photo-, chemo-, and other stimuli

• sensory adaptation occurs by attenuation of the signaling output through reversible methylation of two antagonistically active methylation sites of a transducer

• transducer activation by receptors and transducer methylation determines the signaling output of a cluster in a cooperative manner

• reversible transducer methylation is regulated through feedback via unphosphorylated CheY

Each feature of the model is essential in terms of reproducing transducer demethylation patterns (detected by the release of volatile [methyl-^3^H]-groups as methanol; see Figure 2) and experimental results described for the wildtype and the mutants. The derived mathematical model is based on ordinary differential equations and algebraic equations for equilibrium conditions. In dynamic simulations (e. g., Figure 4), the input was light and the outputs were methanol release and CheYp concentration. Methanol release from cells exposed to different photo- and chemo-stimuli was correlated quantitatively, and the change in CheYp concentration was correlated qualitatively to the motor response in terms of physiological adaptation.

### Adaptation of the CheYp-level in wildtype cells

The initial mathematical model (Model 1) is based on the known molecular components of the phototaxis system and their known photo- and biochemical interactions (see also Additional file 2). Two molecules of sensory rhodopsin I (SRI) form a stable complex (heterotetramer) with two molecules of the SRI-specific transducer (HtrI). The histidine kinase CheA is bound via the scaffold protein CheW to HtrI, which constitutes the R-TWA complex (Figure 1).

The R-TWA complex is assumed to occur in two conformations, active and inactive, which are in thermodynamic equilibrium. Photoexcitation of SRI causes a transient shift in the equilibrium of the two conformations, and the behavioral response of the cell. The behavioral response of the cell in terms of switching the flagellar motor depends on CheA and CheY. CheA autophosphorylates and transfers the phosphate group to CheY. Transducers are methylated by the methyl-transferase CheR and demethylated by the methyl-esterase CheB. Transducers contain one to three methylation sites and the number of sites varies according to the primary structure of the transducer. (Photo-)receptor excitation causes a transient change in transducer demethylation.

The equilibrium of the R-TWA complex depends on the free energy *G *of the active and inactive conformation/state. For thermodynamic reasons, the state with lower free energy is preferred. By direct molecular interaction SRI shifts the equilibrium between the two activity states by changing the free energy of the R-TWA complex depending on the photocycle-state and on the actual activity state of the R-TWA complex. The attractant photointermediate *SRI*_373_, for instance, favors the inactive state by lowering the free energy of the inactive state more compared to the active state (thus ). According to the Boltzmann distribution, the equilibrium probability *A *of being active can be determined (cf. [51-53] and references therein) from the free energy of each possible combination of the photocycle-states of the two SRI proteins with the activity states of the HtrI homodimer

with

As long as the photocycle-state of SRI does not change, the probability of an R-TWA complex of being active depends on the free energy (*F*) in the active and inactive state of the HtrI homodimer and is assumed to be determined by the structural properties of the protein (*F*_0_) as well as by the methylation state of HtrI. Methylated transducers (*Htr*_1_) are assumed to shift the equilibrium towards the active state , whereas unmethylated transducers (*Htr*_0_) shift the equilibrium towards the inactive state :

The demethylation rate (*dm*) is assumed to depend in a proportional manner (symbol ∝) on the conformational state *A *of the R-TWA complex

and the methylation rate (*m*) is assumed to be constant. 

Model 1 produces CheYp-peaks as expected and reversible transducer methylation allows adaptation of the CheYp level (Figure 4A). Spudich and co-workers have shown that activation of HtrI by SRI depends on the interface structure between sensory rhodopsin and its cognate transducer and mutations at or near this interface render wildtype strains into mutants that show an inverted response to orange-light [27,54-56]. The phenotype of such mutants is generated *in silico *in our model by a variation of the free energy changes of the *SRI*_373_-transducer complex such that  (Figure 4A inset).

### Methanol release of wildtype cells and of a cheY-deletion mutant

In simulations, Model 1 failed (Figure 4A) to reproduce the *Halobacterium*-type methanol release pattern and instead, the *E. coli*-type pattern was obtained. This result was insensitive to the numerical values of the rate constants for methylation and demethylation, insensitive to the values of free energy changes upon transducer methylation/demethylation, and insensitive to the rate constants of SRI photocycling measured in wildtype cells. An *E. coli*-type methanol release pattern was obtained no matter whether the transducers in the model carried one or more methylation sites. Moreover, the simulated methanol release pattern remained of the *E. coli*-type, no matter whether (in the model) transducer methylation favors active or inactive conformation, respectively.

In Model 2, we equipped each transducer with two antagonistically behaving methylation sites. We assumed that both sites are accessible to CheB and CheR in both conformational states of the R-TWA complex and that methylation of the one site promotes the active state , whereas methylation of the other site promotes the inactive state . Adaptation then occurs by a shift in the equilibrium of methylation of the activating and the inactivating sites, respectively. Intuitively, one might expect that two antagonistically behaving methylation sites should always cause a net increase in methanol production no matter whether a repulsive or an attractive stimulus is given. However, this is not necessarily the case, as discussed in the following.

Let us assume that both methylation sites,  and , contribute additively to the free energy of the R-TWA complex:

Then, in the simplest case, the rates of demethylation of the activating (*dm*_*A*_) and the inactivating site (*dm*_*I*_) depend linearly on the active conformation *A *and inactive conformation *I*, respectively,

Counterintuitively, numerical simulations show that this model always gives an *E. coli*-type methanol release pattern (Figure 4B). For certain parameter combinations, the methanol release pattern was *E. coli*-type with positive and negative peaks inverted, or no change in the net demethylation rate occurred at all. For approximately linear dependence of the demethylation rates on changes in activity, Δ*A*, the change of demethylation rates for the activating methylation site, Δ*dm*_*A *_∝ Δ*A*, compensates for the changes of demethylation rates of the inactivating methylation site, Δ*dm*_*I *_∝ Δ*I*, and hence no net increase of demethylation occurs in response to attractant or repellent stimulation (Figure 4B). This holds for all tested numerical values of the rate constants for methylation and demethylation.

In a variation of Model 2 (i.e. Model 2 Bp), we assumed that CheA phosphorylates CheB and that CheBp has increased methyl-esterase activity (*k*_*dmB *_> 1):

However, this model was not capable of reproducing the *Halobacterium*-type methanol release patterns (not shown) for all tested numerical values of the rate constants.

It seems that Model 2 is too minimal to be able to reproduce the *Halobacterium*-type demethylation kinetics. Perazzona and Spudich showed that methanol release patterns depend on the presence of the CheY protein [38]. Deletion of the *cheY *gene converts the *Halobacterium*-type methanol release pattern of the wildtype into an *E. coli*-type pattern, indicating that CheY directly or indirectly interacts with the methylation/demethylation system of the transducers. CheY/CheYp might directly interact with the R-TWA complexes, CheB, or CheR through a feedback mechanism, e.g. by alternating the rate of phosphorylation of CheB by CheA.

We found (Figure 4C) that *Halobacterium*-type methanol release patterns are obtained in the simulation of Model 3 if the demethylation rates depend on the transducer activity in a particular nonlinear, e. g. quadratic, manner:

This observation together with the fact that methanol release patterns in *Halobacterium *depend on *cheY *leads to a simple kinetic model for feedback of CheY/CheYp on the transducer demethylation rate (*k*_*dmY *_> 1):

Feedback of CheY/CheYp on the transducer increases the demethylation rates and simultaneously introduces an approximately quadratic dependence of the demethylation rate on the transducer activity (*dm*_*A *_∝ *A*·CheYp ≈ *A*^2 ^and *dm*_*I *_∝ *I*·*CheY *≈ *I*^2^). In the absence of the CheY protein, the rates in the model become approximately linear (e. g., *dm*_*A *_∝ *A*), which explains both, the phenotypes of wildtype (Figure 4C) and of the *cheY *deletion mutant (Figure 4B).

**Figure 5:**
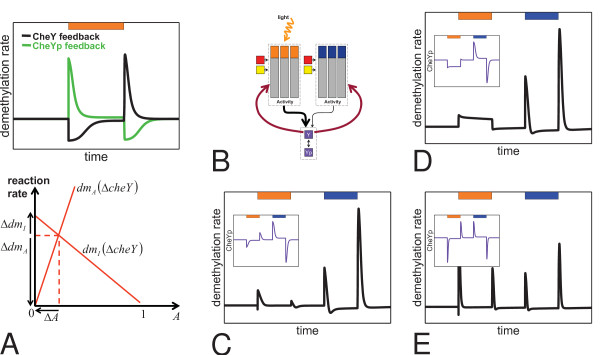
**Analysis of CheY/CheYp-dependent feedback and of different coupling mechanisms**. Orange and blue bars indicate the time intervals of stimulation with attractant orange or, respectively, repellent blue light. **(A) **CheY vs. CheYp dependency of the demethylation rates (top) analyzed in variations of Model 3: only CheY dependency can explain the *cheY *deletion phenotype, as observed in [38]. Explanations of the scheme (bottom) see Text. **(B) **Feedback of stimulated transducers via CheY on the demethylation rates of unstimulated transducers. **(C) **In a wildtype version of the model (two methylation-efficient transducer populations) *Halobacterium*-type methanol release patterns and adapting CheYp levels (inset) are obtained. **(D) **Model 4 with methylation-deficiencies on the transducers mediating orange light responses, and normal methylation on transducers mediating blue light responses: adaptation of CheYp (inset) is defect and the *Halobacterium*-type methanol release is not obtained on stimulation of the methylation-deficient transducers. **(E) **R-TWA complexes are conformationally coupled (see also Figure 6) in Model 5. Adaptation of CheYp (inset) and methanol release is comparable to the wildtype as observed experimentally.

Demethylation and methylation rates in Equation (1) depend on CheY and CheYp concentration, respectively. Hence the model suggests that CheY regulates demethylation of both methylation sites depending on its phosphorylation state and depending on the activity of the R-TWA complex (feedback mechanism 1: CheY and CheYp). However, an alternative kinetic mechanism yields a similar behavior of the system, namely if only the unphosphorylated form of the CheY protein inhibits demethylation of the activating methylation site and simultaneously enhances demethylation of the inactivating methylation site (feedback mechanism 2: CheY), e. g.:

Here, inhibition and activation is modeled by kinetics of the Michaelis-Menten-type. Yet another and similar regulatory mechanism results if only CheYp modulates the demethylation rates (feedback mechanism 3: CheYp), e. g.:

Feedback mechanisms 2 and 3, i. e. regulation of demethylation through the unphosphorylated or the phosphorylated form of the CheY protein, respond differently to the genetic deletion of CheY. If the modification rates were regulated by CheY only (feedback mechanism 2), then *cheY *deletion (concentration CheY = 0) would increase *dm*_*A *_to *A*·(1 + *k*_*dmY*_) and, contrarily, would decrease *dm*_*I *_to *I*. The resulting steady state equilibrium is then shifted towards lower activity of the R-TWA complexes (Figure 5A). A drop of activity down to zero (saturating attractant, *A *= 0) would then cause an overall drop of demethylation. Upon saturating stimulation with repellents (*A *= 1), an overall increase of demethylation would occur (Figure 5A), as experimentally observed in the *cheY *deletion mutant [38]. Dependency on CheYp alone (feedback mechanism 3) would produce the opposite methanol release pattern (Figure 5A), in contradiction to the experimental observations. Thus, in the model, the unphosphorylated form of CheY (rather than CheYp) is the active component in regulating transducer demethylation at the two antagonistically active sites.

The remaining possibility (feedback mechanism 1; Equation (1)) is that both CheY and CheYp regulate demethylation of the two sites, however in a reciprocal manner. The steady-state would then be barely shifted in a *cheY *deletion mutant. Parameters can be found that reproduce the *Halobacterium*-type methanol release patterns. However, the resulting model turned out to be very sensitive to parameter variations when rate constants were fitted to experimental data. In addition to not being robust with respect to parameter variations, the model is not minimal, i. e. would need more parameters and interactions to explain the data. We therefore continue with the simplest model in which demethylation of the two sites is only regulated by the unphosphorylated form of the CheY protein.

Accordingly, feedback by the CheY protein (feedback mechanism 2) was incorporated into Model 3 to give Model 3 fbm2 and the effect of different numerical values of the reaction rates was analyzed. Parameter combinations that produced *Halobacterium*-type methanol release patterns were easily obtained (cf. Figure 4C), while positive and negative peaks of CheYp were obtained as expected.

The fact that CheB in *H. salinarum *has a CheY-like domain (that may become phosphorylated by CheA) may lead to alternative kinetic models in which CheB/CheBp-feedback is responsible for producing the *Halobacterium*-type methanol release patterns. If both CheB and CheY compete for phosphate from CheA, then the absence of the CheY-protein could cause an increased phospho-transfer to CheB. If, in addition, CheBp has increased demethylation activity (cf. Model 2 Bp), then this would result in a higher (transient) demethylation rate for repellent stimuli, and a lower (transient) demethylation rate for attractant stimuli. The methanol release in a *cheY *deletion mutant would then be of the *E. coli*-type, as experimentally observed.

To analyze the possibility of a CheB/CheBp-based mechanism, we assumed CheB and CheBp-dependent demethylation rates (see also Figure 3) of the form

The additional parameters *k*_1 _≥ 1 and *k*_2 _≥ 1 were introduced to account for possible cooperative effects. For an appropriate choice of the parameters *k*_1 _and *k*_2_, CheB and CheBp introduce a nonlinear dependence on activity/inactivity which may be approximative quadratic - similar as for CheY/CheYp in Equation (1) - and may then produce the *Halobacterium*-type pattern observed in the wildtype. We found parameters for models (Model 3B fbm4 and Model 3B fbm5) with a cubic (*k*_2 _= 3) dependence on CheB/CheBp for which the wildtype methanol release pattern could be reproduced. However, only Model 3B fbm4 with a linear (*k*_1 _= 1) dependence on activity/inactivity could predict the *E. coli*-type methanol release patterns of the *cheY *deletion mutant. We did not favor the possibility of a CheB/CheBp-based feedback mechanism (see also Discussion), because the wildtype/*Halobacterium*-type methanol-release patterns are still observed in a *B. subtilis *mutant in which the CheY-like domain of CheB is deleted [46].

### Adaptation of cells with methylation-deficient HtrI

Cells expressing HtrI with all putative methylation sites disabled by site-directed mutagenesis are able to respond and to adapt to light, and transiently release methanol as observed in the wildtype [38]. Since Model 3 fbm2 failed to reproduce this experimental finding, it had to be extended to a model in which excitation of the SRI-HtrI complex is communicated to a different transducer type with different substrate specificity, which in turn is reversibly demethylated. Presumptive mechanisms of functional coupling might be conformational coupling of different transducer types, or feedback by modulating the methyl-esterase and methyl-transferase activity at transducers other than the actually stimulated transducers.

Extending Model 3 fbm2 by an additional, non-stimulated and methylation-efficient transducer type (activity *A*_*u*_) yields Model 4. Looking at wildtype cells, now two different R-TWA populations are coupled via CheY that feeds back on both transducer types (Figure 5B). Due to the conformationally independent but functionally coupled populations, the overall activity is given by the linear superposition/weighted mean of both activities

where *k*_*u *_and *k*_*s *_represent the contributions of unstimulated (*A*_*u*_) and stimulated (*A*_*s*_) transducers due to different expression levels.

In the wildtype case, parameters for simulating *Halobacterium*-type methanol release with adaptation of the CheYp level were found (Figure 5C). However, when simulating the model with one methylation-deficient transducer population and one transducer population where reversible methylation is normal, we could not find any set of parameters that reproduced the experimentally observed wildtype *Halobacterium*-type methanol release pattern and adaptation (Figure 5D). As the activity of the unstimulated transducers stays constant and as the mutated, stimulated transducers cannot be methylated, any change of the demethylation rates in the model can only occur through feedback by CheY the concentration of which is changed by the stimulated transducers. Then the demethylation rates become linear

and cannot explain the *Halobacterium*-type methanol release (cf. Figure 4B). Hence, in the framework of this model, feedback by CheY alone cannot be the only mechanism that mediates cross-talk between stimulated and non-stimulated transducers. Phosphorylation of CheB through the stimulated transducers and an increased demethylation of the non-stimulated transducers by phosphorylated CheB could provide such a cross-talk mechanism. However, we found no parameters in such a model (Model 4 Bp) that predicted the *Halobacterium*-type methanol release pattern of the non-methylatable HtrI mutant (not shown). The missing additional mechanism could be conformational coupling among different transducer types/species within a (signaling) unit, and this is considered in Model 5.

In Model 5, all R-TWA complexes within a unit contribute to the overall activity in a nonlinear and cooperative manner via the phototaxis transducers and cognate photoreceptors, via the binding state of the chemotaxis transducers/receptors, and via the methylation state of all transducers within the unit. By formulating the free energy changes of every possible state of the R-TWA signaling unit, the probability of being active can be derived from the Boltzmann distribution in a similar manner as for the SRI-HtrI heterotetramer. Suitable parameter values for simulating experimentally observed methanol release patterns and adaptation of the CheYp level for wildtype cells, CheY-deficient or non-methylatable HtrI mutants were easily obtained (Figure 5E).

### Fitting of the final model to quantitative experimental data

**Figure 6:**
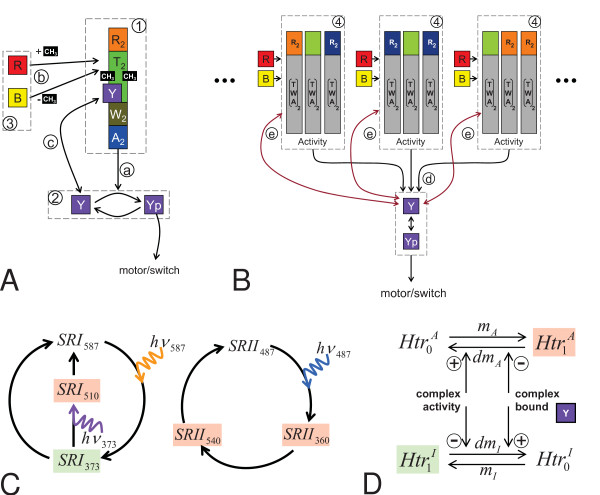
**Overview of mechanisms and interactions between components in the final Model 6**. The same colors as in Figure 1 are used for identical proteins in **(A) **and **(B)**. For short notation the prefix "Che" is omitted. Activating and inactivating signaling states are shaded in red and green, respectively. Model components and interactions in **(A) **and **(B)**: (1) R-TWA complex; (2) diffusive response regulator CheY/CheYp; (3) reversible transducer methylation by CheB and CheR; (4) R-TWA units of different transducer/receptor types; the active conformation (corresponding to phosphorylated CheA) of the R-TWA complex (1) enhances the phosphate transfer (a) to CheY; signals of all R-TWA complexes are integrated (d) by CheY phosphorylation; CheY binds (c) to the R-TWA complexes and globally coordinates reversible transducer methylation (e); each R-TWA signaling unit (4) consists of several (here three) R-TWA complexes of different receptors/transducers. CheYp binds to the motor and increases switching probability. **(C) **SRI is the sensor for orange and near-uv light, SRII senses blue light. Photon absorption produces a sequence of intermediate photoproducts where the long-lived photointermediates are signaling states. Other, short-lived photointermediates are not shown. **(D) **Regulation of antagonistic reversible transducer demethylation/methylation by bound CheY and by complex activity during the adaptation process. Notation: , unmethylated/methylated activating site; , unmethylated/methylated inactivating site. Upon a saturating repellent (increase of activity *A *and no unbound CheY) transducer modification is shifted towards demethylation of the activating site () which brings the system back into an adapted state.

**Figure 7:**
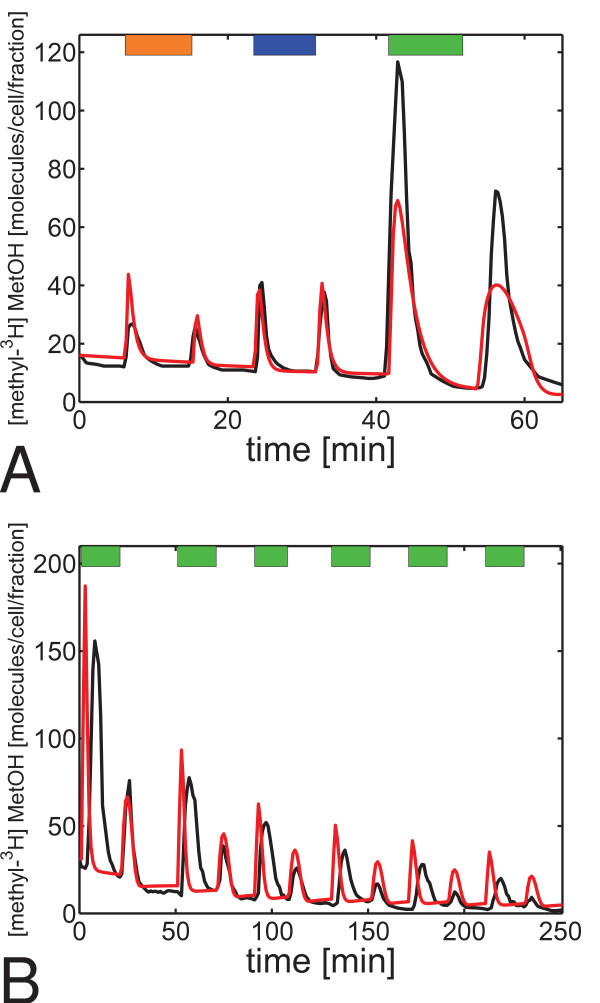
**Comparison of simulation and quantitative experimental data**. Orange bars indicate stimulation with attractant orange light, blue bars stimulation with repellent blue light, and green stimulation with attractant chemo-stimuli. The final model was fitted (see Methods) to quantitative experimental data from References [37] in **(A)**, and [35] in **(B)**. The output of the model is shown in red, experimental data in black. The same parameter values were used for all fittings, but only the parameter describing the experimental side conditions (see Methods and Additional file 1) were varied to account for different experimental conditions and strains. In the simulations, 3500 methyl-groups were radio-labeled in **(B) **at the start of the experiment, which correlates well with the minimum number (2800) of tactically active methyl-groups determined by [35] and suggests that parameters were chosen reasonably.

Each feature of Model 5 is essential in the sense that its omission produces a model that contradicts one of the experimental observations. To validate whether Model 5 is able to reproduce experimental data quantitatively, we had to take into account the influence of the measurement technique used and extended the model by radio-labeling and methanol release kinetics (see Figure 2 and Methods). The final model (Model 6; see Figure 6) was fitted to the results of flow assay experiments (Figure 2) and it quantitatively reproduced experimental data (Figure 7).

## Discussion

We presented the first quantitative, predictive model of excitation and adaptation in halobacterial phototaxis and chemotaxis which explains the methanol release patterns in response to attractant and to repellent stimulation in the wildtype, in a *cheY *deletion mutant, and in a mutant in which the stimulated transducer species is methylation-deficient. Essential elements of our model are two antagonistically active methylation sites, demethylation rates that depend on feedback by CheY, and conformationally coupled heterologous receptors/transducers assembled in cooperative signaling units.

The model integrates proposed mechanistic features both from *E. coli *and *B. subtilis*, the combination of which allows to quantitatively reproduce the experimental data on stimulus-induced methanol release in *Halobacterium*, once the appropriate parameter values are chosen. Features from *E. coli *are cross-talk of heterologous transducer species by direct conformational coupling within a (signaling) unit (modeled by a Monod-Wyman-Changeux-type model of cooperativity) [51-53,57,58]. Features from *B. subtilis *are two methylation sites on the transducers that behave antagonistically in response to attractant and to repellent stimulation, and a feedback of CheY/CheYp on reversible receptor methylation [43,44,46,59].

Feedback of CheY on transducer demethylation is based on direct experimental evidence by [38] who showed that the normal *Halobacterium*-type methanol release pattern (a positive peak in response to both attractant and repellent stimulation) is converted into an *E. coli*-like pattern (a negative peak in response to attractant stimulation and positive in response to repellent). However, feedback by CheY was not sufficient to explain the experimental finding that release of methanol is caused by stimulation of a mutant transducer in which all putative methylation sites have been experimentally deleted [38]. Within the cooperative unit of heterologous receptors/transducers, methylation deficiencies as well as different equipment with methylation sites (some transducers have 1, others have 3 methylation sites [31,38]) are compensated allowing the mutant to adapt.

An alternative potential mechanism for feedback might perhaps occur by reversible phosphorylation of the response regulator domain on CheB [33]. In *E. coli*, methyl-esterase activity of CheB is increased by phosphorylation of the response regulator domain [39], which presumably forms a feedback loop *in vivo *that controls the basal activity and the speed of motor response [60]. Phosphorylation of CheB in *E. coli *is also considered to increase robustness of adaptation [61] in chemotaxis, while it seems not to be required for precise adaptation [62] for which receptor conformation dependent demethylation is sufficient [63]. In *B. subtilis*, CheB also has a phosphorylation domain but the role of CheB-phosphorylation is not known in detail. A mutant strain with deleted CheB response regulator domain still produces the identical methanol release patterns, albeit at a generally higher turn-over/basal-level [46]. Thus, the methanol release in response to attractant and to repellent stimulation is independent of CheB phosphorylation in *B. subtilis *[46].

As we have no experimental evidence for stimulus-dependent CheB phosphorylation in *Halobacterium*, we have computationally analyzed the effect of CheB phosphorylation by assuming that methyl-esterase activity of CheB is increased by phosphorylation. CheB phosphorylation could not reproduce the wildtype methanol-release pattern for linear demethylation rates (Model 2 Bp), nor could CheB phosphorylation mimic the effects mediated by cooperative transducer interaction in terms of methanol release caused by stimulation through a non-methylatable mutant transducer (Model 4 Bp). Furthermore, in a extension of the final Model 6 by CheB-phosphorylation, *Halobacterium*-type methanol release patterns were still obtained, which suggests that CheB phosphorylation does not interfere with the proposed kinetic mechanisms of our model.

We have also analyzed the possibility that the *Halobacterium*-type methanol release patterns are generated by a CheB/CheBp-based feedback mechanism (Model 3B fbm4 and Model 3B fbm5). We found parameters in Model 3B fbm4 that predict the patterns of the wildtype and of the *cheY *deletion. In any case, it is not possible by an analysis of the mathematical models to exclude or disprove a direct or indirect role of CheB phosphorylation in methylation-dependent adaptation. To discriminate experimentally between a CheY-based and a CheB/CheBp-based feedback mechanism, we propose to measure the methanol release patterns of a mutant in which the CheY-like domain of CheB has been deleted. If *Halobacterium*-type patterns are still obtain in such a mutant, then this would suggest that CheB-phosphorylation is not part of the feedback-mechanism which generates the *Halobacterium*-type patterns. Since the *Halobacterium*-type patterns are still observed in a corresponding *B. subtilis *mutant [46], we did not favor the CheB/CheBp-based feedback mechanisms.

Additional feedback-loops of methylation independent adaptation processes such as in *B. subtilis *via the interplay of CheD, CheC and CheYp [10,47,59] may further contribute to adaptation in *Halobacterium*, but we have no experimental evidence for this. We included enzymatic hydrolysis of CheYp by a CheC phosphatase in a variation of Model 6 to analyze the potential effect of a phosphatase on methanol release and CheYp levels. Again, for a wide range of parameters the additional mechanism of CheC-phosphatase did not interfere with or destroyed the *Halobacterium*-type patterns qualitatively or quantitatively. As expected due to the proposed phosphatase activity of CheC, the level of CheYp in the adapted state was significantly lower as predicted by the model lacking CheC. For repellents, the CheYp level was severalfold higher compared to the adapted level.

Experimental results regarding stimulus-induced changes in transducer methylation in *Halobacterium *measured by gel fluorography as published by different authors are contradictory [35,36,38] and have therefore not been taken into account for our model. Instead, we focused on reliable, reproducible, and temporally resolved quantitative data on the demethylation rates as measured with the flow assay. However, for the simplest case of constant methylation rates (Model 6), our model suggests that the transducer methylation level initially drops in response to all kind of stimuli and subsequently slowly returns to the pre-stimulus level, as it was experimentally observed in *B. subtilis *[45].

Parameters in the final model were fitted to experimental data from different groups. Parameters from *E. coli *and *B. subtilis *([59,64] and references therein) served as starting values and a parameter set was found that quantitatively reproduced the experimental data. Further parameter optimizations or accounting for yet unmodeled mechanistic details (e. g., aberrant number of methylation sites, tactically non-active methylation sites, different enzymatic activity of CheB and CheR at different methylation sites, and coordination of antagonistic methylation by CheC and CheD) might improve fitting results further, but additional model extensions are not sufficiently supported by currently available experimental data in *H. salinarum*.

## Conclusions

We provided a kinetic model for signal processing in photo- and chemotaxis in the archaeon *H. salinarum *suggesting an essential role of receptor cooperativity, antagonistic reversible methylation, and a CheY-dependent feedback on transducer demethylation.

Further iterative cycles of experiments and mathematical modeling are required to reduce the number of undetermined parameters and to incorporate more mechanistic details.

Nevertheless, the predictive computational model and the parameter set obtained now allows to infer CheYp concentration changes upon excitation and adaptation and to correlate these changes to the measurable response of the flagellar motor switch. This will lead to a data-based coherent model of excitation, adaptation, and motor response in halobacterial phototaxis.

## Methods

### Protein homology modeling

Protein modeling was done with the SWISS MODEL server [65,66] using the alignment interface mode. Inputs were sequence alignments produced with BioEdit version 7.0.9.0 [67] and the ClustalW algorithm [68]. The HaloLex server [69,70], the UniProt server [71], and the Protein Data Bank server [72] were used to retrieve protein sequences of halobacterial and homologous genes. Templates for homology modeling were homologues from *Escherichia coli *(serine chemotaxis receptor [PDB:1QU7] for the cytosolic part of HtrI), *Salmonella typhimurium *(CheA P1 domain [PDB:1I5N:A]; CheR [PDB:1AF7:A]; CheB [PDB:1A2O:A]), *Thermotoga maritima *(CheA P2 domain [PDB:1U0S:A]; CheA P3-P5 domains [PDB:1B3Q:A]; CheW1 [PDB:1K0S:A]; CheY [PDB:1U0S:Y]; CheD [PDB:2F9Z:D]; CheC1 and CheC2 [PDB:1XKR:A]), and *Natronomonas pharaonis *(transmembrane part of SRII and HtrII [PDB:1H2S]). The graphic representations of PDB files of models generated by SWISS MODEL were produced with the molecular visualization software RasTop [73].

### Implementation of the models

The models were implemented in the form of ordinary differential equations and the reaction rates were modeled by mass-action kinetics in most cases. In multi-protein-complexes, rates also depend on certain states of the complex, such as activity *A*, methylation state, or bound CheY in the R-TWA complex. To keep the number or reactions and parameters low, we did not model all intermediate complexes and we also assumed quasi-steady-state for some reactions. Explicit formulation of mass-action kinetics and modeling intermediate complexes did not change the qualitative and quantitative behavior of the models to any significant degree. Simulations were performed in MatLab 7.6 (Mathworks, U.S.) with the Systems Biology Toolbox 2.0 [74] on a standard laptop PC. Simulation scripts are available, see Additional file 3.

### Model parameter estimation and fitting to experimental data

Parameters of the SRI and SRII photocycles (including thermal decay rates, quantum yield, absorption cross section) and copy numbers of the retinal proteins were taken from the literature [7,75,76]. Copy numbers of chemotaxis proteins were determined by mass-spectrometry (M. Schlesner, private communication). Parameters of *B. subtilis *and *E. coli *models (see [59] and references therein) and data from quantitative and qualitative experiments (see Additional file 2) were sufficient to provide starting values for a fitting of Model 6 to quantitative, experimental data on transient methanol release.

### Description of Model 6

Expressions above or below the reaction arrows are the corresponding reaction rates used. Parameters are given in the tables of the Additional file 1.

#### SRI and SRII photoreceptors

Interconversions between the photointermediates (*SRI*_587_, *SRI*_373_) of the orange and uv light photoreceptor SRI were modeled by the following set of reactions:

with

Note that we did not model uv light excitation.

Interconversions between the photointermediates (*SRII*_487_, *SRII*_360_, *SRII*_540_) of the blue light photoreceptor SRII were modeled by:

with

#### Chemotaxis transducers

The dynamics of ligand (*Lig*) binding to the binding protein BasB of the chemotaxis transducer BasT (detection of branched chain amino acids: leucine, isoleucine, valine, methionine and cysteine [13]) was not explicitly modeled and quasi-steady-state was assumed instead. The amount of ligand bound to BasB was given by:

#### R-TWA unit excitation

The model assumes that each R-TWA unit consists of *N*_*tot *_R-TWA complexes. Thus, a R-TWA signaling unit has 2·*N*_*tot *_transducer proteins (and an according number of receptor proteins), 2·*N*_*tot *_CheW proteins, and 2·*N*_*tot *_CheA proteins (see Figure 1). The stoichiometry of different receptor-transducer types in the R-TWA unit is given by their expression levels relative to the total number of transducer proteins *Htr*_*tot*_:

with

Due to conformational coupling, the R-TWA complexes in the cooperative unit are at the same time either all active or all inactive. The equilibrium probability of being active, *A*, followed from a Monod-Wyman-Changeux (MWC)-type model for cooperative chemotaxis-receptor complexes [51-53]:

with

The equilibrium probability of being active in the absence of ligand or light, *F*, depends on the methylation state of the R-TWA unit.

Note, the assumptions made by the MWC model on the allosteric regulatory mechanisms in an oligomer do not exactly translate to phototaxis receptors/transducers complexes, because each receptor molecule can only interact with the transducer molecule to which it is stably bound. The MWC model should therefore be considered as a continuous approximation of the actual discrete, stochastic process in which also ligand depletion plays a crucial role (S. Streif, unpublished). However, for saturating stimuli the approximation error is negligible. We used the approximate MWC model to allow building a deterministic model consisting of a system of ordinary differential and algebraic equations that can be solved by numerical integration.

#### R-TWA unit adaptation

In the model, adaptation is performed by methylation and demethylation of the 2·2·*N*_*tot *_methylation sites on the transducers in a R-TWA unit, whereof 2·*N*_*tot*_sites are activating, and 2·*N*_*tot *_are inactivating. An aberrant number of methylation sites did not change the qualitative results and were consequently not considered. Notation of methylation states: , unmethylated/methylated activating site; , unmethylated/methylated inactivating site. Methylation alters the activity of the R-TWA unit:

CheY binds to the R-TWA complex (*TWA*)

R-TWA complex bound CheY (*TWAY*) regulates demethylation by kinetics of the Michaelis-Menten type:

Methylation and demethylation reactions were modeled by:

with

Due to conservation relationships it follows:

#### Two-component system

Phosphorylation of the histidine kinase CheA and phosphate-group transfer to the response regulator CheY was modeled by

with

CheYp is hydrolyzed in a first-order reaction

#### Radio-labeling and methanol release kinetics in the flow assay

The time scale of methanol release adaptation is longer than for physiological adaptation because of the effects of mixing time in the apparatus (Figure 2). The mixing time in the apparatus extends the time course of response to addition and removal of a stimulating chemical compound [40] and on the measurements of [methyl-^3^H]-methyl-groups (superscript 3*H*).

Cells are incubated in the presence of [methyl-^3^H]-methionine during experiment preparations. The methanol release rate strongly depends on the experimental conditions such as the start of the measurements after incubation, and the extent of [methyl-^3^H]-methionine uptake varies between different strains, days and experiments [38]. We introduced parameters to account for the varying experimental conditions:  and  give the fraction of the intracellular methionine (*Met*_*tot*_) and, respectively, the fraction of the transducers that are actually [methyl-^3^H]-labeled at the start of the experiments.

The total methionine storage (*Met*_*tot*_) was assumed constant, but the cellular storage of [methyl-^3^H]-methionine (*Met*^3*H*^) degradates with a first order kinetics [35] due to diffusion and in exchange with extracellular methionine:

[methyl-^3^H]-groups are transferred to the transducers by methylation, which additionally exploits *Met*^3*H*^. Each methylation site can be [methyl-^3^H]-unlabeled ( and ) and [methyl-3H]-labeled ( and ) and each state was explicitly modeled. Demethylation of a [methyl-^3^H]-labeled methylation site leads to [methyl-^3^H]-methanol () production and which is released by the cells. The methylation and demethylation rates were accordingly modified, e. g. for the active methylation site:

with

Due to the flow of  through the apparatus, the actually measured  is delayed (time constant *T*_*out *_= *T*_*flow*_).

 is collected and sampled in fractions of *T*_*S *_seconds length, which in the model corresponds to

The output of Model 6 (*fraction*_*i*_, *i *∈ {1, 2, 3, ...}) was plotted together with the experimental data in Figure 7. ¿From the experimental data [35,37], the number of released molecules of [methyl-^3^H]-methanol per fraction and cell was determined using a specific activity of 70-80 Ci/mmol of [methyl-^3^H]-methionine in the cells [34-36], and a counting efficiency of 0.5 [37].

Chemotaxis-transducer ligand is subjected to the flow delay *T*_*in *_= *T*_*flow *_(Figure 2), which was modeled by

where *Lig*_*in *_is the input stimulus and *Lig *is the ligand concentration in the flow chamber that is actually sensed by the cells. Any delay (*T*_*delay*_, see Figure 2) due to the flow was taken into account in the simulations by delaying the onset of the stimuli. The mixing time has no influence on the kinetics for photostimuli [35].

## Abbreviations

SRI: sensory rhodopsin I (subscripts indicate the absorption maximum of the corresponding photointermediate, e. g. *SRI*_587_); SRII: sensory rhodopsin II (subscripts indicate the absorption maximum of the corresponding photointermediate, e. g. *SRII*_487_); Htr: halobacterial transducer protein (subscripts indicate the methylation state); CheA/CheAp: unphosphorylated/phosphorylated autohistidine kinase; CheB/CheBp: unphosphorylated/phosphorylated methyl-esterase; CheR: methyl-transferase; CheC: phosphatase of CheY, used interchangeably for the three CheC's (CheC1, CheC2, CheC3); CheW: scaffolding protein of the R-TWA complex, used interchangeable for the two CheWs (CheW1, CheW2); CheY/CheYp: unphosphorylated/phosphorylated diffusible response regulator; ec: extra cellular; ic: intra cellular; MetOH: methanol; dm: demethylation rate; m: methylation rate; R-TWA complex: receptor-transducer-CheW-CheA oligomer; R-TWA (signaling) unit: several R-TWA complexes constituting a cooperative (signaling) unit in which all complexes are either all active or all inactive; fbm: feedback mechanism; uv: (near-)ultraviolet.

## Authors' contributions

SS, WM and DO contributed to model design, analysis and interpretation of the data. SS performed the modeling and simulations. SS and WM drafted the manuscript. SS, WM and DO revised the manuscript. All authors read and approved the final manuscript.

## Supplementary Material

Additional file 1 **Parameters of Model 6**. The PDF-file contains the values and a descriptions for each parameter. Click here for file

Additional file 2 **Quantitative and qualitative findings relevant to the model**. The PDF-file contains additional references and a summary of the quantitative and qualitative findings that were relevant to the model. Click here for file

Additional file 3 **Scripts for simulation of Models 1-6**. Simulation of the models requires MatLab http://www.mathworks.com and the Systems Biology Toolbox 2 [74]. To run Model 1 - Model 5 use simModels_1to5.m and use simModel_6.m for Model 6. Click here for file
