# Supplementary material for: A predictive computational model of the kinetic mechanism of stimulus-induced transducer methylation and feedback regulation through CheY in archaeal phototaxis and chemotaxis
Source: BMC Syst Biol. 2010 Mar 18;4:27. doi: 10.1186/1752-0509-4-27 (PMC2857822; doi:10.1186/1752-0509-4-27)
Supplement: Additional file 1 — Parameters of Model 6. The PDF-file contains the values and a descriptions for each parameter. [file 1752-0509-4-27-S1.PDF]

Table 1 - Parameters of Model 6

Symbol	Value [unit]	Description
σ_{587}	1.94e-20 [m^2]	Absorption cross section
ϕ_{SRI}	0.4	Quantum yield
k^d_{373}	0.924 [1/s]	Thermal decay
I_{587}	See Tab. 2 [photons/m^2/s]	Intensity of orange light
σ_{487}	1.81e-20 [m^2]	Absorption cross section
ϕ_{SRII}	0.64	Quantum yield
k^d_{360}	0.577 [1/s]	Thermal decay
k^d_{540}	0.21 [1/s]	Thermal decay
I_{487}	See Tab. 2 [photons/m^2/s]	Intensity of blue light
Lig_{in}	See Tab. 2	Ligand concentration for chemotaxis stimuli
N_{tot}	8	Number of R-TWA complexes in a R-TWA unit
$K^A_{587} = K^I_{587}$	1.5	Equilibrium constant for SRI_{587} in active/inactive state
K^A_{373}	1.5e-2	Equilibrium constant for SRI_{373} in active state
K^I_{373}	1.5e-3	Equilibrium constant for SRI_{373} in inactive state
$K^A_{487} = K^I_{587}$	0.8	Equilibrium constant for $SRII_{487}$ in active/inactive state
$K^A_{360} = K^A_{540}$	0.8e-3	Equilibrium constant for $SRII_{360}$ and $SRII_{540}$ in active state
$K^I_{360} = K^I_{540}$	0.8	Equilibrium constant for $SRII_{360}$ and $SRII_{540}$ in inactive state
K^I_{BasT}	4e-6	BasT equilibrium constant in the inactive state
K^A_{BasT}	4	BasT equilibrium constant in the active state
K^M_{BasB}	5e-1	Michaelis-Menten constant of BasB/ligand binding
k^p_A	1 [1/s]	CheA auto-phosphorylation
k^p_{AY}	10 [1/s]	Phospho-transfer from CheAp to CheY
k^h_Y	3.333 [1/s]	First-order rate constant of CheYp hydrolysis
F_0	1	Equilibrium constant in the absence of ligand and methylation
ΔG^A_1	-15	Free energy change (set dimension free) due to methylation of the activating methylation site
ΔG^I_1	15	Free energy change (set dimension free) due to methylation of the inactivating methylation site
k^a_{TWAY}	1e-3 [1/s]	Association of CheY and R-TWA complexes
k^d_{TWAY}	100 [1/s]	Dissociation of CheY and R-TWA complexes
k^m	1e-4 [1/s]	CheY-independent methylation rate constant
k^{dm}	2e-4 [1/s]	CheY-independent demethylation rate constant
k^{dm}_{YA}	1.2e-2 [1/s]	CheY-dependent methylation rate constant
k^{dm}_{YI}	3.2e-1 [1/s]	CheY-dependent demethylation rate constant
T_{flow}	See Tab. 2 [s]	First-order time delay in the flow apparatus
Htr^{3H}_{frac}	See Tab. 2	Fraction of [methyl-^{3}H]-labeled transducers
Met^{3H}_{frac}	See Tab. 2	Fraction of [methyl-^{3}H]- methionine
k^d_{Met}	5.023e-5 [1/s]	1st-order time constant of wash-out of the intracellular [methyl-^{3}H]-labeled methionine storage
Met_{tot}	1.2e5 [1/cell]	Total amount of labeled and unlabeled methionine [37]
Htr_{tot}	10000 [1/cell]	Transducer molecules
$HtrI_{tot}$, SRI_{tot}	1500 [1/cell]	SRI and HtrI molecules

$HtrII_{tot}$, $SRII_{tot}$	800 [1/cell]	SRII and HtrII molecules
$BasB_{tot}$, $BasT_{tot}$	4000 [1/cell]	BasT (chemotaxis transduer) molecules
$CheA_{tot}$	10000 [1/cell]	CheA molecules
$CheY_{tot}$	3000 [1/cell]	CheY molecules
T_S	See Tab. 2 [s]	Sample collection time in the flow assay

Table 2 - Experiment dependent parameters for quantitative simulations of Model 6

Phototaxis/chemotaxis stimuli differed in intensity/concentration between the experiments. Furthermore, due to different experimental procedures in different labs and by different experimenters, different strains and cell densities, and varying methionine uptake on different days [38], we had to adjust some parameters to account for the varying side conditions in the experiments.

Figure	Ref.	I_{587}	I_{487}	Lig_{in}	T_S	T_{flow}	Htr^{3H}_{frac}	Met^{3H}_{frac}	Cells	Strains
Figure 7A	[37]	1.17e21	1.9e20	5e-5	18	37.5	0.6	0.3	9e8	Flx37
Figure 7B	[35]	-	-	1e-7	60	57.5	0.35	0.2	5e8	Flx15
