# Supplementary material for: A predictive computational model of the kinetic mechanism of stimulus-induced transducer methylation and feedback regulation through CheY in archaeal phototaxis and chemotaxis
Source: BMC Syst Biol. 2010 Mar 18;4:27. doi: 10.1186/1752-0509-4-27 (PMC2857822; doi:10.1186/1752-0509-4-27)
Supplement: Additional file 2 — Quantitative and qualitative findings relevant to the model. The PDF-file contains additional references and a summary of the quantitative and qualitative findings that were relevant to the model. [file 1752-0509-4-27-S2.PDF]

References

1. Alam M, Oesterhelt D: **Morphology, function and isolation of halobacterial flagella.** *J Mol Biol* 1984, **176**(4):459–475.
2. Streif S, Staudinger WF, Marwan W, Oesterhelt D: **Flagellar rotation in the archaeon *Halobacterium salinarum* depends on ATP**. *J Mol Biol* 2008, **384**:1–8.
3. Marwan W, Alam M, Oesterhelt D: **Rotation and switching of the flagellar motor assembly in *Halobacterium halobium***. *J Bacteriol* 1991, **173**(6):1971–1977.
4. Streif S, Staudinger WF, Oesterhelt D, Marwan W: **Quantitative analysis of signal transduction in motile and phototactic cells by computerized light stimulation and model based tracking**. *Rev Sci Instrum* 2009, **80**(2):023709, [http://link.aip.org/link/?RSI/80/023709/1].
5. Spudich JL, Stoeckenius W: **Photosensory and chemosensory behavior of *Halobacterium halobium* (phototaxis, chemotaxis, bacteriorhodopsin, halobacteria)**. *Photobioch Photobiop* 1979, **1**:43–53.
6. Stoeckenius W, Wolff EK, Hess B: **A rapid population method for action spectra applied to *Halobacterium halobium***. *J Bacteriol* 1988, **170**(6):2790–2795.
7. Hoff WD, Jung KH, Spudich JL: **Molecular mechanism of photosignaling by archaeal sensory rhodopsins**. *Annu Rev Biophys Biomol Struct* 1997, **26**:223–258, [http://dx.doi.org/10.1146/annurev.biophys.26.1.223].
8. Marwan W, Bibikov SI, Montrone M, Oesterhelt D: **Mechanism of photosensory adaptation in *Halobacterium salinarium***. *J Mol Biol* 1995, **246**(4):493–499.
9. Falke JJ, Bass RB, Butler SL, Chervitz SA, Danielson MA: **The two-component signaling pathway of bacterial chemotaxis: a molecular view of signal transduction by receptors, kinases, and adaptation enzymes.** *Annu Rev Cell Dev Biol* 1997, **13**:457–512, [http://dx.doi.org/10.1146/annurev.cellbio.13.1.457].
10. Rao CV, Glekas GD, Ordal GW: **The three adaptation systems of *Bacillus subtilis* chemotaxis.** *Trends Microbiol* 2008, **16**(10):480–487, [http://dx.doi.org/10.1016/j.tim.2008.07.003].
11. Borkovich KA, Alex LA, Simon MI: **Attenuation of sensory receptor signaling by covalent modification**. *Proc Natl Acad Sci U S A* 1992, **89**(15):6756–6760.
12. Hess JF, Bourret RB, Simon MI: **Histidine phosphorylation and phosphoryl group transfer in bacterial chemotaxis**. *Nature* 1988, **336**(6195):139–143, [http://dx.doi.org/10.1038/336139a0].
13. Kokoeva MV, Storch KF, Klein C, Oesterhelt D: **A novel mode of sensory transduction in archaea: binding protein-mediated chemotaxis towards osmoprotectants and amino acids.** *EMBO J* 2002, **21**(10):2312–2322, [http://dx.doi.org/10.1093/emboj/21.10.2312].
14. Rudolph J, Oesterhelt D: **Chemotaxis and phototaxis require a CheA histidine kinase in the archaeon *Halobacterium salinarium*.** *EMBO J* 1995, **14**(4):667–673.
15. Pfeiffer F, Schuster SC, Broicher A, Falb M, Palm P, Rodewald K, Ruepp A, Soppa J, Tittor J, Oesterhelt D: **Evolution in the laboratory: the genome of *Halobacterium salinarum* strain R1 compared to that of strain NRC-1.** *Genomics* 2008, **91**(4):335–346, [http://dx.doi.org/10.1016/j.ygeno.2008.01.001].
16. Alexander RP, Zhulin IB: **Evolutionary genomics reveals conserved structural determinants of signaling and adaptation in microbial chemoreceptors.** *Proc Natl Acad Sci U S A* 2007, **104**(8):2885–2890, [http://dx.doi.org/10.1073/pnas.0609359104].
17. Szurmant H, Ordal GW: **Diversity in chemotaxis mechanisms among the bacteria and archaea**. *Microbiol Mol Biol Rev* 2004, **68**(2):301–319, [http://dx.doi.org/10.1128/MMBR.68.2.301-319.2004].
18. Schlesner M: **The *Halobacterium salinarum* taxis signal transduction network: a protein-protein interaction study**. *PhD thesis*, Ludwig-Maximilians-Universität München 2008.
19. Gestwicki JE, Lamanna AC, Harshey RM, McCarter LL, Kiessling LL, Adler J: **Evolutionary conservation of methyl-accepting chemotaxis protein location in Bacteria and Archaea.** *J Bacteriol* 2000, **182**(22):6499–6502.
20. Oesterhelt D, Marwan W: **Change of membrane potential is not a component of the photophobic transduction chain in *Halobacterium halobium*.** *J Bacteriol* 1987, **169**(8):3515–3520.
21. Krah M, Marwan W, Oesterhelt D: **A cytoplasmic domain is required for the functional interaction of SRI and HtrI in archaeal signal transduction.** *FEBS Lett* 1994, **353**(3):301–304.
22. Krah M, Marwan W, Verméglio A, Oesterhelt D: **Phototaxis of *Halobacterium salinarium* requires a signalling complex of sensory rhodopsin I and its methyl-accepting transducer HtrI.** *EMBO J* 1994, **13**(9):2150–2155.
23. Zhang XN, Zhu J, Spudich JL: **The specificity of interaction of archaeal transducers with their cognate sensory rhodopsins is determined by their transmembrane helices**. *Proc Natl Acad Sci U S A* 1999, **96**(3):857–862.
24. Ferrando-May E, Krah M, Marwan W, Oesterhelt D: **The methyl-accepting transducer protein HtrI is functionally associated with the photoreceptor sensory rhodopsin I in the archaeon *Halobacterium salinarium*.** *EMBO J* 1993, **12**(8):2999–3005.
25. Chen X, Spudich JL: **Demonstration of 2:2 stoichiometry in the functional SRI-HtrI signaling complex in *Halobacterium* membranes by gene fusion analysis.** *Biochemistry* 2002, **41**(12):3891–3896.
26. Zhang XN, Spudich JL: **HtrI is a dimer whose interface is sensitive to receptor photoactivation and His-166 replacements in sensory rhodopsin I.** *J Biol Chem* 1998, **273**(31):19722–19728.
27. Sasaki J, Spudich JL: **Signal transfer in haloarchaeal sensory rhodopsin – transducer complexes**. *Photochem Photobiol* 2008, **84**(4):863–868, [http://dx.doi.org/10.1111/j.1751-1097.2008.00314.x].

28. Sineshchekov OA, Sasaki J, Phillips BJ, Spudich JL: **A Schiff base connectivity switch in sensory rhodopsin signaling.** *Proc Natl Acad Sci U S A* 2008, **105**(42):16159–16164, [http://dx.doi.org/10.1073/pnas.0807486105].

29. Gordeliy VI, Labahn J, Moukhametzianov R, Efremov R, Granzin J, Schlesinger R, Büldt G, Savopol T, Scheidig AJ, Klare JP, Engelhard M: **Molecular basis of transmembrane signalling by sensory rhodopsin II-transducer complex.** *Nature* 2002, **419**(6906):484–487, [http://dx.doi.org/10.1038/nature01109].

30. Moukhametzianov R, Klare JP, Efremov R, Baeken C, Göppner A, Labahn J, Engelhard M, Büldt G, Gordeliy VI: **Development of the signal in sensory rhodopsin and its transfer to the cognate transducer.** *Nature* 2006, **440**(7080):115–119, [http://dx.doi.org/10.1038/nature04520].

31. Koch MK, Staudinger WF, Siedler F, Oesterhelt D: **Physiological sites of deamidation and methyl esterification in sensory transducers of *Halobacterium salinarum*.** *J Mol Biol* 2008, **380**:285–302.

32. Koch MK, Oesterhelt D: **MpcT is the transducer for membrane potential changes in *Halobacterium salinarum*.** *Mol Microbiol* 2005, **55**(6):1681–1694, [http://dx.doi.org/10.1111/j.1365-2958.2005.04516.x].

33. Rudolph J, Tolliday N, Schmitt C, Schuster SC, Oesterhelt D: **Phosphorylation in halobacterial signal transduction.** *EMBO J* 1995, **14**(17):4249–4257.

34. Nordmann B, Lebert MR, Alam M, Nitz S, Kollmannsberger H, Oesterhelt D, Hazelbauer GL: **Identification of volatile forms of methyl groups released by *Halobacterium salinarium*.** *J Biol Chem* 1994, **269**(23):16449–16454.

35. Alam M, Lebert M, Oesterhelt D, Hazelbauer GL: **Methyl-accepting taxis proteins in *Halobacterium halobium*.** *EMBO J* 1989, **8**(2):631–639.

36. Lebert M: **Methylierung und Methylthiolierung bei *Halobacterium salinarium*.** *PhD thesis*, Ludwigs-Maximilians-Universität München 1991.

37. Nordmann B: **Bedeutung der Methylierung und Methylthiolierung in *Halobacterium salinarium* und Identifizierung neuer Gene für signalübertragende Proteine.** *PhD thesis*, Ludwig-Maximilians-Universität München 1994.

38. Perazzona B, Spudich JL: **Identification of methylation sites and effects of phototaxis stimuli on transducer methylation in *Halobacterium salinarum*.** *J Bacteriol* 1999, **181**(18):5676–5683.

39. Lupas A, Stock J: **Phosphorylation of an N-terminal regulatory domain activates the CheB methylesterase in bacterial chemotaxis.** *J Biol Chem* 1989, **264**(29):17337–17342.

40. Kehry MR, Doak TG, Dahlquist FW: **Stimulus-induced changes in methylesterase activity during chemotaxis in *Escherichia coli*.** *J Biol Chem* 1984, **259**(19):11828–11835.

41. Sundberg SA, Alam M, Lebert M, Spudich JL, Oesterhelt D, Hazelbauer GL: **Characterization of *Halobacterium halobium* mutants defective in taxis.** *J Bacteriol* 1990, **172**(5):2328–2335.

42. Brooun A, Bell J, Freitas T, Larsen RW, Alam M: **An archaeal aerotaxis transducer combines subunit I core structures of eukaryotic cytochrome c oxidase and eubacterial methyl-accepting chemotaxis proteins.** *J Bacteriol* 1998, **180**(7):1642–1646.

43. Zimmer MA, Tiu J, Collins MA, Ordal GW: **Selective methylation changes on the *Bacillus subtilis* chemotaxis receptor McpB promote adaptation.** *J Biol Chem* 2000, **275**(32):24264–24272, [http://dx.doi.org/10.1074/jbc.M004001200].

44. Zimmer MA, Szurmant H, Saulmon MM, Collins MA, Bant JS, Ordal GW: **The role of heterologous receptors in McpB-mediated signalling in *Bacillus subtilis* chemotaxis.** *Mol Microbiol* 2002, **45**(2):555–568.

45. Kirby JR, Saulmon MM, Kristich CJ, Ordal GW: **CheY-dependent methylation of the asparagine receptor, McpB, during chemotaxis in *Bacillus subtilis*.** *J Biol Chem* 1999, **274**(16):11092–11100.

46. Bunn MW, Ordal GW: **Receptor conformational changes enhance methylesterase activity during chemotaxis by *Bacillus subtilis*.** *Mol Microbiol* 2004, **51**(3):721–728.

47. Muff TJ, Ordal GW: **The CheC phosphatase regulates chemotactic adaptation through CheD.** *J Biol Chem* 2007, **282**(47):34120–34128, [http://dx.doi.org/10.1074/jbc.M706432200].

48. Staudinger W: **Investigations on flagellar biogenesis, motility and signal transduction of *Halobacterium salinarum*.** *PhD thesis*, Ludwig-Maximilians-Universität München 2008.

49. Muff TJ, Ordal GW: **The diverse CheC-type phosphatases: chemotaxis and beyond.** *Mol Microbiol* 2008, **70**(5):1054–1061, [http://dx.doi.org/10.1111/j.1365-2958.2008.06482.x].

50. Nutsch T, Oesterhelt D, Gilles ED, Marwan W: **A quantitative model of the switch cycle of an archaeal flagellar motor and its sensory control.** *Biophys J* 2005, **89**(4):2307–2323, [http://www.biophysj.org/cgi/content/abstract/89/4/2307].

51. Sourjik V, Berg HC: **Functional interactions between receptors in bacterial chemotaxis.** *Nature* 2004, **428**(6981):437–441, [http://dx.doi.org/10.1038/nature02406].

52. Monod J, Wyman J, Changeux JP: **On the nature of allosteric transitions: a plausible model.** *J Mol Biol* 1965, **12**:88–118.

53. Mello BA, Tu Y: **An allosteric model for heterogeneous receptor complexes: understanding bacterial chemotaxis responses to multiple stimuli.** *Proc Natl Acad Sci U S A* 2005, **102**(48):17354–17359, [http://dx.doi.org/10.1073/pnas.0506961102].

54. Jung KH, Spudich JL: **Suppressor mutation analysis of the sensory rhodopsin I-transducer complex: insights into the color-sensing mechanism.** *J Bacteriol* 1998, **180**(8):2033–2042.

55. Olson KD, Zhang XN, Spudich JL: **Residue replacements of buried aspartyl and related residues in sensory rhodopsin I: D201N produces inverted phototaxis signals.** *Proc Natl Acad Sci U S A* 1995, **92**(8):3185–3189.

56. Sasaki J, Phillips BJ, Chen X, Eps NV, Tsai AL, Hubbell WL, Spudich JL: **Different dark conformations function in color-sensitive photosignaling by the sensory rhodopsin I-HtrI complex.** *Biophys J* 2007, **92**(11):4045–4053, [http://dx.doi.org/10.1529/biophysj.106.101121].

57. Keymer JE, Endres RG, Skoge M, Meir Y, Wingreen NS: **Chemosensing in *Escherichia coli*: two regimes of two-state receptors.** *Proc Natl Acad Sci U S A* 2006, **103**(6):1786–1791, [http://dx.doi.org/10.1073/pnas.0507438103].

58. Endres RG, Oleksiuk O, Hansen CH, Meir Y, Sourjik V, Wingreen NS: **Variable sizes of *Escherichia coli* chemoreceptor signaling teams.** *Mol Syst Biol* 2008, **4**:211, [http://dx.doi.org/10.1038/msb.2008.49].

59. Rao CV, Kirby JR, Arkin AP: **Design and diversity in bacterial chemotaxis: a comparative study in *Escherichia coli* and *Bacillus subtilis*.** *PLoS Biol* 2004, **2**(2):E49, [http://dx.doi.org/10.1371/journal.pbio.0020049].

60. Hauri DC, Ross J: **A model of excitation and adaptation in bacterial chemotaxis.** *Biophys J* 1995, **68**(2):708–722, [http://dx.doi.org/10.1016/S0006-3495(95)80232-8].

61. Kollmann M, Løvdok L, Bartholomé K, Timmer J, Sourjik V: **Design principles of a bacterial signalling network.** *Nature* 2005, **438**(7067):504–507, [http://dx.doi.org/10.1038/nature04228].

62. Alon U, Surette MG, Barkai N, Leibler S: **Robustness in bacterial chemotaxis.** *Nature* 1999, **397**(6715):168–171, [http://dx.doi.org/10.1038/16483].

63. Barkai N, Leibler S: **Robustness in simple biochemical networks.** *Nature* 1997, **387**(6636):913–917, [http://dx.doi.org/10.1038/43199].

64. Morton-Firth CJ, Shimizu TS, Bray D: **A free-energy-based stochastic simulation of the Tar receptor complex.** *J Mol Biol* 1999, **286**(4):1059–1074, [http://dx.doi.org/10.1006/jmbi.1999.2535].

65. SWISS MODEL server: *[http://swissmodel.expasy.org]*[http://swissmodel.expasy.org]. [Accessed 2 Oct 2008].

66. Schwede T, Kopp J, Guex N, Peitsch MC: **SWISS-MODEL: An automated protein homology-modeling server.** *Nucleic Acids Res* 2003, **31**(13):3381–3385.

67. Hall TA: **BioEdit: a user-friendly biological sequence alignment editor and analysis program for Windows 95/98/NT**. *Nucleic Acids Symp Ser* 1999, **41**:95–98.

68. Thompson JD, Higgins DG, Gibson TJ: **CLUSTAL W: improving the sensitivity of progressive multiple sequence alignment through sequence weighting, position-specific gap penalties and weight matrix choice.** *Nucleic Acids Res* 1994, **22**(22):4673–4680.

69. HaloLex server: *[http://www.halolex.mpg.de]*[http://www.halolex.mpg.de]. [Accessed 2 Oct 2008].

70. Pfeiffer F, Broicher A, Gillich T, Klee K, Mejía J, Rampp M, Oesterhelt D: **Genome information management and integrated data analysis with HaloLex.** *Arch Microbiol* 2008, **190**(3):281–299, [http://dx.doi.org/10.1007/s00203-008-0389-z].

71. UniProt server: *[http://www.uniprot.org]*[http://www.uniprot.org]. [Accessed 2 Oct 2008].

72. Protein Data Bank server: *[http://www.rcsb.org]*[http://www.rcsb.org]. [Accessed 2 Oct 2008].

73. RasTop: *[http://www.geneinfinity.org/rastop]*. [Version 2.2].

74. Schmidt H, Jirstrand M: **Systems Biology Toolbox for MATLAB: a computational platform for research in systems biology.** *Bioinformatics* 2006, **22**(4):514–515, [http://dx.doi.org/10.1093/bioinformatics/bti799].

75. Bogomolni RA, Spudich JL: **The photochemical reactions of bacterial sensory rhodopsin-I. Flash photolysis study in the one microsecond to eight second time window.** *Biophys J* 1987, **52**(6):1071–1075.

76. Otomo J, Marwan W, Oesterhelt D, Desel H, Uhl R: **Biosynthesis of the two halobacterial light sensors P480 and sensory rhodopsin and variation in gain of their signal transduction chains**. *J Bacteriol* 1989, **171**(4):2155–2159.

77. Kentner D, Sourjik V: **Spatial organization of the bacterial chemotaxis system.** *Curr Opin Microbiol* 2006, [http://dx.doi.org/10.1016/j.mib.2006.10.012].

78. Wadhams GH, Armitage JP: **Making sense of it all: bacterial chemotaxis**. *Nat Rev Mol Cell Biol* 2004, **5**(12):1024–1037, [http://dx.doi.org/10.1038/nrm1524].

79. Marwan W, Oesterhelt D: **Signal formation in the halobacterial photophobic response mediated by a fourth retinal protein (P480).** *J Mol Biol* 1987, **195**(2):333–342.

80. Takahashi T, Yan B, Spudich JL: **Sensitivity increase in the photophobic response of *Halobacterium halobium* reconstituted with retinal analogs: a novel interpretation for the fluence-response relationship and a kinetic modeling.** *Photochem Photobiol* 1992, **56**(6):1119–1128.

81. Yan B, Spudich J: **Evidence that the repellent receptor form of sensory rhodopsin I is an attranctant signaling state**. *Photochem Photobiol* 1991, **54**(6):1023–1026.

82. Kokoeva MV, Oesterhelt D: **BasT, a membrane-bound transducer protein for amino acid detection in *Halobacterium salinarum*.** *Mol Microbiol* 2000, **35**(3):647–656.

83. Aregger M: **Charakterisierung von *cheW*-Deletionsmutanten in *Halobacterium salinarum*.** *PhD thesis*, Ludwig-Maximilians-Universität München 2003.

84. Rudolph J, Oesterhelt D: **Deletion analysis of the *che* operon in the archaeon *Halobacterium salinarium*.** *J Mol Biol* 1996, **258**(4):548–554, [http://dx.doi.org/10.1006/jmbi.1996.0267].

85. Spudich EN, Takahashi T, Spudich JL: **Sensory rhodopsins I and II modulate a methylation/demethylation system in *Halobacterium halobium* phototaxis.** *Proc Natl Acad Sci U S A* 1989, **86**(20):7746–7750.

86. Hildebrand E, Schimz A: *Sensing and Response in Microorganisms*, Elsevier Science Publisher B.V. 1985 chap. Behavioral Pattern and its Photosensory Control in *Halobacterium halobium*.

Table 3 - Quantitative and qualitative findings relevant to the model

Finding	Reference	See Figure
SRI "senses" orange and uv light and SRII blue light; SRI_{510}, $SRII_{360}$, and $SRII_{540}$ are repellent signaling states; SRI_{373} is an attractant signaling state	[75, 79–81]	6C
BasB is the binding protein for the chemotaxis transducer BasT and senses leucine, isoleucine, valine, methionine and cysteine	[82]	6B
SRI and SRII interact with their cognate transducers HtrI and HtrII through a stable complex; HtrI forms homodimers; SRI and HtrI form tetramers at a 2:2 stoichiometry	[22, 24–26]	1; 6A (1)
Photoactivation of SRI causes a conformational change in its transducer HtrI; the SRI-HtrI complex exists in two conformations; the equilibrium of the complex is determined by photointermediates, methylation and by mutations at or near the interface; some mutations lead to inverted phototaxis responses	[27–30, 54–56]	1; 4A; 6A (1); 6C
CheA and CheW are essential for halobacterial photo- and chemotaxis; CheA and CheW physically interact with the transducers; CheA is an autophosphorylating histidine kinase	[14, 18, 33, 48, 83, 84]	1; 6A (1)
A diffusible response regulator that is subjected to decay transmits the signal from the polarly localized receptors to the switch; CheA is dephosphorylated by CheY; deletion of *cheA* or *cheY* result in a complete loss of photophobic response	[14, 20, 33, 38, 48, 84]	6A (a) (1) (2); 6B
Spontaneous hydrolysis of phosphorylated CheY is very rapid (*in vitro*); estimated half-life below $\leq 5\,s$	[33]	6A (2)
H. salinarum has homologous (*cheC1*, *cheC2*, *cheC3*) of the CheY phosphatase CheC in *B. subtilis*, and no homologous of other known phosphatases like FliY, CheX, or CheZ	[15, 48, 49]	-
Sensory adaptation of the motor response occurs by switching off the signaling activity of receptor molecules	[8]	6A (3)(b)
Both chemotaxis and phototaxis signals are integrated and modulate the excitation and adaptation systems	[5, 35, 85]	6B
Htr's are methylated by CheR and demethylated by CheB; CheB has a CheY-like domain	[31–33]	6A (3) (b)
18 orthologous methyl-accepting taxis proteins (Htr's) are found in the genome of *H. salinarum*; 12 of the 18 transducers contain 1-3 methylation sites per molecule	[15, 31]	6A (1); 6B
Methyl-accepting proteins localize in clusters and preferentially at the cell poles	[19]	6B
Step-like attractive and repulsive stimuli both cause a transient increase in methanol release rate; the methanol release adapts to constant stimuli and return roughly to the pre-stimulus level; the brief period of altered rate of release of volatile methyl-groups corresponds to the brief period of behavioral adaptation after photo-stimulation	[34–37]	7
Sensory adaptation to attractant and to repellent stimulation through SRI and stimulus-induced methanol release (as in wildtype) occurs even if the methylation sites of HtrI are deleted; deletion of *htrI* abolishes any methylation response with respect to SRI specific stimuli	[38]	4A; 5D; 6B
Deletion of *cheY* transforms the *Halobacterium*-type demethylation pattern into an *E. coli*-type pattern	[38]	4
Behavioral adaptation to photostimuli is completed after one switching interval ($\leq 20\,s$ saturating repellent and $\leq 50\,s$ saturating attractant)	[5, 86]	-
Less than 100 methyl-groups are released upon step-like stimuli of saturating (attractant) orange or (repellent) blue light; at least 1800-3500 methyl-groups are released upon a pulse sequence of saturating (attractant) chemo-stimuli	[34–36]	7
Methanol release is qualitatively similar but quantitatively different for phototaxis and chemotaxis stimuli: effects of photo-stimuli differed from those caused by chemo-stimuli in magnitude (approx. 5% of the magnitude of a chemo-stimulus-induced release) and duration (< 1 min versus several minutes for chemo-stimuli); Chemo-stimuli are subjected to turbulent flow/mixing kinetics in the flow experiment	[34–36]	2; 7
4000 SRI, 430 SRII, 12000 CheA, 12000 CheW's and 3000 CheY molecules per cell	[18, 76]	-
